# Multimodal EEG–EMG and FEM-Based Adaptive Control of Passive Upper-Limb Exoskeletons

**DOI:** 10.3390/s26123924

**Published:** 2026-06-20

**Authors:** Luigi Bibbò, Filippo Laganà, Salvatore A. Pullano, Giovanni Angiulli

**Affiliations:** 1 Department of Civil, Energy, Environment and Materials (DICEAM), “Mediterranea” University of Reggio Calabria, I-89124 Reggio Calabria, Italy; 2 Laboratory of Biomedical Applications Technologies and Sensors (BATS), Department of Health Science, “Magna Græcia” University of Catanzaro, 88100 Catanzaro, Italy; filippo.lagana@unicz.it (F.L.); pullano@unicz.it (S.A.P.); 3 DIIES Department, Mediterranea University, I-89122 Reggio Calabria, Italy

**Keywords:** passive exoskeleton, EEG–EMG, multimodal fusion, adaptive control, cognitive load, finite element modeling (FEM), CNN–LSTM, brain–machine interface (BMI)

## Abstract

Integrating neural and muscular signals into wearable robotics enables adaptive assistance during real-world tasks. This study proposes a multimodal neural interface for passive exoskeletons that combines electroencephalography (EEG) and electromyography (EMG) signals to classify motor gestures and estimate real-time cognitive and muscular effort, supported by finite-element-based biomechanical modeling. The system was implemented on the Ottobock Shoulder X passive exoskeleton© and validated using synchronous EEG–EMG acquisition via the LiveAmp platform©, a commercially available platform that was not developed specifically for this study. A hybrid CNN–LSTM architecture with deep fusion was employed to enhance robustness and responsiveness under realistic operating conditions. This study proposes a multimodal neural interface for the software-level adaptive assistance of passive upper-limb exoskeletons. While the physical device maintains a static mechanical profile, the proposed digital framework achieves adaptation by interpreting the user’s physiological and motor states. Ten healthy participants performed three functional tasks (screwing, moving the box, and lifting the box) under five assistive conditions. Finite element modeling (FEM) was used to characterize the torque–angle relationship of the passive exoskeleton and to support the interpretation of experimentally observed assistive torque profiles. The FEM model, used as an offline biomechanical analysis tool to aid in the interpretation of experimental results, has not been integrated into the real-time control loop. Results showed an average classification accuracy of 90%, an F1-score of 0.85, and inference latency below 180 ms, confirming real-time applicability. Cognitive indices such as the Cognitive Load Index (CLI) and Frontal Asymmetry Index (FAI) enabled adaptive modulation of assistance strategies without requiring active actuation, thereby preserving the device’s intrinsic passive nature. Comparative torque analysis highlighted the ergonomic benefits of passive systems in mid-range postures, while Finite Element Method (FEM) supported analysis clarified their limitations under highly dynamic loads compared to active solutions. These findings advance multimodal brain–machine interfaces for wearable robotics by integrating physiological sensing, deep learning, and biomechanical modeling, offering a safe, energy-efficient, and adaptive approach with potential rehabilitation, occupational ergonomics, and human–robot applications.

## 1. Introduction

Wearable robotic technologies are no longer confined to highly controlled laboratory settings. They are being tested and deployed in rehabilitation, occupational ergonomics, and care settings that mirror everyday use. In this context, upper-limb exoskeletons are a major focus of research because of their potential to reduce physical strain, support motor rehabilitation, and lower the incidence of work-related musculoskeletal disorders [1,2,3]. Despite significant technological advances over the past decade, translating exoskeletons into practice remains challenging. Concerns about adaptability, usability, and acceptance have been the primary barriers to their widespread application beyond the laboratory [4]. From a design perspective, upper-limb exoskeletons can be classified as either active or passive. Active exoskeletons incorporate powered actuators to generate adjustable assistive torques that can be tuned to task demands and to inferred user intent [5,6]. This flexibility is matched by increased weight, power demand, mechanical and control complexity, and the need to meet safety and certification standards [7]. Passive exoskeletons, on the other hand, use elastic or quasi-elastic components, such as springs and cam mechanisms, to redistribute loads and compensate for gravity [8,9]. This approach is attractive for its inherent safety and simplicity, which have contributed to its growing popularity and use in industrial ergonomics and early rehabilitation [10]. Accordingly, the assistance provided by the device is entirely determined by its mechanical design, while the proposed framework aims to enhance how that assistance is used rather than how it is physically generated. However, the inability to adapt to changing user conditions and task demands has been a major drawback, especially in domains characterized by task variability and mental fatigue. A distinction must be made between physical actuation and adaptive behavior. The term ‘adaptive’ does not imply the generation of an active response but refers to the modulation of interaction strategies and the interpretation of the user’s intentions within the limits of a purely passive mechanical system. Although passive exoskeletons cannot generate active torque, adaptive strategies can be implemented to detect, interpret, and optimize user-specific assistance without altering the underlying mechanical structure [11,12]. In this context, adaptive assistance refers to modulating the effective support provided by the passive exoskeleton through user-state interpretation, without introducing powered actuation or external energy input. To mitigate these limitations, increasing attention has been directed toward integrating neural and myoelectric sensing modalities to enable adaptive behavior in wearable robotic systems [13]. While these methods provide insights into cognitive and cortical processes, they often require substantial calibration and are sensitive to task context and movement-related artifacts. On the other hand, surface electromyography (EMG) measures muscle activation, which is closely related to physical execution [14]. Recent evidence further suggests that muscle mass distribution and neuromuscular coordination significantly influence movement efficiency and motor control strategies, reinforcing the relevance of EMG-derived information for adaptive human–machine interaction systems [15]. Thus, the combined approach of EEG and EMG has been suggested to overcome the limitations of the individual methods when used alone [16,17,18]. Multimodal EEG–EMG fusion enables simultaneous observation of central and peripheral aspects of motor behavior, yielding a more informative representation of the human state during interaction with wearable devices [19]. Methodologically, different fusion strategies have been explored. Early approaches rely on feature-level concatenation, whereas late fusion schemes combine the outputs of independent classifiers [20,21]. Recently, deep learning methods have been integrated to address the complex relationships between modalities. Convolutional neural networks (CNNs), commonly used for feature extraction, and recurrent neural networks with long short-term memory (LSTM) units have been used to model temporal relationships [22,23]. Hybrid models that combine CNNs and LSTMs have been reported to improve the robustness of the approach over traditional machine learning methods. However, some recent contributions have focused mainly on the classification approach’s accuracy [24]. Nevertheless, inference time and efficiency are important considerations for wearable systems used in unconstrained environments [25]. EEG-derived indices, including the Cognitive Load Index (CLI) and the Frontal Asymmetry Index (FAI), have been proposed as quantitative measures of mental workload and emotional engagement [26]. These indices, widely used individually in their respective fields, are limited in their integration into real-time support systems due to challenges with robustness and interpretability in dynamic environments [27,28]. Although these metrics have been validated in controlled experiments, they are most often used for offline assessment and are rarely incorporated into adaptive assistance strategies. At the same time, cognitive loads and affective states have been increasingly recognized as important determinants of human performance and safety during assisted tasks [29]. Finite element modeling (FEM) has therefore gained traction as a tool for analyzing mechanical behavior [30], load transfer, and ergonomic performance in exoskeleton systems [31,32]. Despite its potential, FEM is typically employed as an offline design or validation step and is seldom integrated with physiological sensing or adaptive control frameworks, limiting its effectiveness in real-time applications and responsiveness in exoskeleton systems [33,34]. Addressing these gaps, the present work introduces an integrated framework for passive upper-limb exoskeletons that combines multimodal EEG–EMG sensing, deep learning-based task classification, cognitive load estimation, and FEM-supported mechanical analysis. Thus, the proposed approach, which integrates the physiological, cognitive, and biomechanical aspects of the exoskeleton system within a unified framework, is expected to improve the responsiveness and interpretability of exoskeleton-assisted performance while preserving the inherent safety and mechanical simplicity of the approach. To avoid ambiguity, this study explicitly states that no semi-active or active actuation is introduced. The Ottobock Shoulder X is used in its original commercial configuration and remains fully passive throughout all experiments. Any adaptation is limited to software-level interpretation of the user’s cognitive and motor state and does not modify the mechanical assistance provided by the passive elastic elements. A practical example of this adaptive paradigm occurs during repetitive overhead tasks. When elevated cognitive workload or muscular fatigue is detected through EEG–EMG monitoring, the system does not modify the exoskeleton’s torque output but instead provides contextual feedback to operators or supervisors, supports task-scheduling decisions, and enables more accurate interpretation of the user’s biomechanical condition. Therefore, the adaptive benefit arises from improved human–system interaction rather than from active mechanical intervention.

## 2. Materials and Methods

This section outlines the materials, modeling strategies, experimental methods, and computational framework used to develop and validate the proposed EEG–EMG-based adaptive control system for passive shoulder exoskeletons. The methodological approach integrates finite element biomechanical modeling, synchronous multimodal neurophysiological acquisition, and deep learning-based multimodal classification to provide ergonomic, safe, and cognitively aware assistance during real-world motor tasks.

### 2.1. Finite Element Biomechanical Model

**Figure 1:**
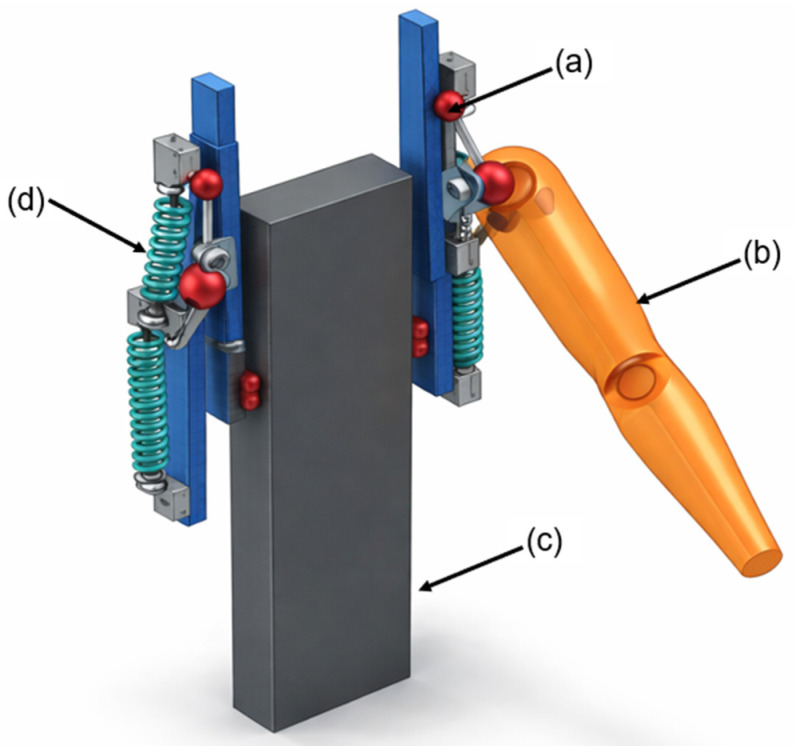
Geometry representation of the passive shoulder exoskeleton and the simplified upper-limb model.

The first phase presents a finite-element biomechanical model of the passive shoulder exoskeleton in COMSOL Multiphysics 5.2© to quantify torque assistance, joint loads, and mechanical behavior across the full range of shoulder motion. The finite element model was developed exclusively as an offline biomechanical framework to analyse torque–angle relationships, load distribution, and structural response under different postural conditions. The model was not integrated into the real-time adaptive assistance pipeline but was instead used to support the interpretation of experimental and physiological results within a physics-based biomechanical context [35]. FEM analysis enables the characterization of torque–angle relationships, load distribution, and structural response under different postural conditions. This reinforces the interpretative nature, from a biomechanical perspective, of the observed assistive effects, whilst maintaining the computational efficiency of the adaptive model. The FEM geometry includes a simplified representation of the upper limb, as shown in Figure 1, along with passive exoskeleton components such as cables, springs, and attachment interfaces.

Figure 1 identifies the main biomechanical components of the system: (a) the shoulder joint, (b) the arm segment, (c) the torso structure, and (d) the rear supporting frame.

**Figure 2:**
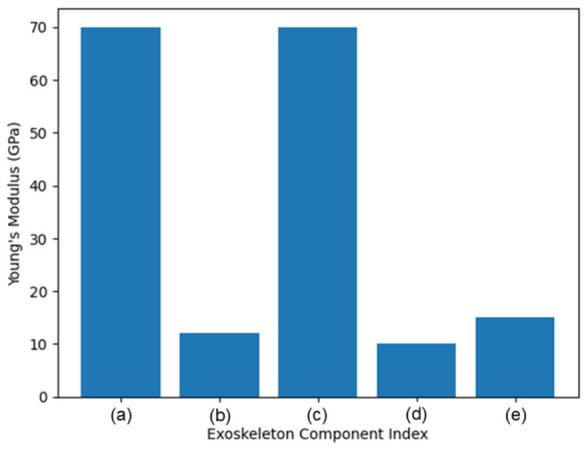
Finite element geometry of the passive shoulder exoskeleton and the simplified upper-limb model.

The exoskeleton is modelled as a deformable mechanical structure to evaluate internal stresses and elastic energy accumulation, while the upper limb is represented as a rigid body with a single degree of freedom at the shoulder joint. This modeling approach isolates the effect of elastic assistance while accounting for realistic inertial and gravitational effects [36]. Figure 1 also clarifies the positional relationship between the rear frame and the articulated parts attached to the arm, which is important for the load-transfer mechanism. Material properties are assigned in line with those of typical lightweight assistive devices. Figure 2 shows the distribution of elastic moduli for the exoskeleton’s main functional components.

High stiffness values correspond to aluminium load-bearing elements, whereas lower values indicate polymer-reinforced compliant components. As shown in Figure 2, components (a) and (c) have high stiffness values (around 70 GPa), consistent with aluminium load-bearing elements, whereas components (b), (d), and (e) exhibit lower elastic moduli (10–15 GPa), typical of polymer-reinforced materials. The assignment of material properties across the components was not uniform but intentionally differentiated to reflect their distinct functional roles within the exoskeleton, as shown in Table 1.

**Figure 3:**
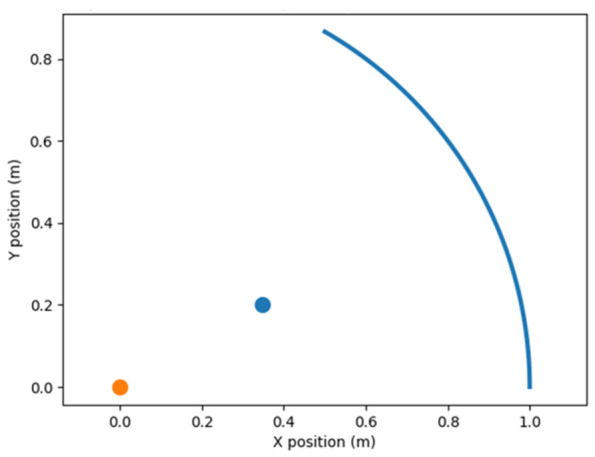
Equivalent rigid-body model of the upper arm.

This stiffness heterogeneity reflects a deliberate design strategy that combines structural rigidity where load transmission is required with compliance where deformation and comfort are functionally beneficial. This design rationale aligns with established principles in wearable robotics, where the balance between stiffness and compliance is key to optimizing both mechanical performance and user comfort. The elastic modulus values assigned to each component were selected based on standard engineering material properties reported in the literature and the manufacturer’s datasheets. Specifically, the elastic modulus of aluminium alloys was selected from standard engineering databases (e.g., the ASM Handbook), where typical values range from 68 to 72 GPa. The mechanical properties of fibre-reinforced polymer components were instead derived from the literature on glass- and carbon-reinforced composites, which generally exhibit elastic moduli of 8–20 GPa, depending on fibre content and orientation. Similar elastic modulus ranges have been reported in recent investigations on polymer-based biomechanical and wearable structures, confirming their suitability for assistive-device modeling [37,38]. Accordingly, the selected range of 10–15 GPa represents a conservative and realistic approximation for compliant structural elements in wearable assistive devices [39]. To further support the reliability of these assumptions, the FEM results were qualitatively compared with experimentally observed torque–angle trends reported for comparable passive exoskeleton systems, such as the Ottobock Shoulder X, showing consistent mechanical behavior. These choices are consistent with standard practices in biomechanical FEM of assistive devices. For further evaluation of robustness, a sensitivity analysis was done by changing the values of elastic modulus within ±10%. It turned out that there were no significant differences in stress distribution, deformation modes, and torque–angle behavior; thus, the chosen biomechanical model proved to be reliable even under material parameter uncertainties. The upper limb is modelled as an equivalent rigid body introduced exclusively to account for inertial and gravitational loading [40]. Its kinematic and mass representation is illustrated in Figure 3.

The arm is simplified to a single rigid segment rotating about the shoulder joint, with the center of mass located at 40% of the upper-arm length from the joint. The curved trajectory shown in the figure represents the path the arm follows during shoulder elevation. Figure 3 shows that the model captures the essential biomechanical behavior of the limb while avoiding unnecessary anatomical complexity, allowing the focus to remain on the interaction between the exoskeleton and the arm. The geometry of the cable–spring transmission system defines the kinematic relations governing elastic-element deformation [41]. The cable attachment points, transmission path, and geometric parameters are used to compute the cable length as a function of the shoulder elevation angle *θ*.

As shoulder elevation increases, the transmission path lengthens, producing an elongation ∆*l(θ)* of the elastic elements. The same geometry also defines the effective lever arm *r(θ)*, which determines how the spring force is converted into torque about the shoulder joint. Assuming linear elastic behavior and frictionless transmission, the passive assist torque generated by the exoskeleton is expressed as (1).

(1) $$\tau_{p}\left(\theta \right)=k_{s}∆l\left(\theta \right)r(\theta )$$

where *k_s_* is the spring’s equivalent stiffness. Formulation (1) is derived from Hooke’s law applied to the cable–spring mechanism, assuming linear elastic behavior and negligible friction. The geometric relationship between cable elongation and joint angle is determined by the transmission system’s kinematic configuration.

The gravitational torque generated by the upper limb is modelled by (2).

(2) $$\tau_{g}\left(\theta \right)=m_{a}gl_{cm}\sin\left(\theta \right)$$

where *m_a_* denotes the equivalent mass of the upper-arm segment and *l_cm_* represents the distance between the shoulder joint and the arm centre of mass. Expression (2) follows standard rigid-body biomechanical modeling, in which the arm is approximated as a single segment with mass concentrated at its center of mass. The net torque that must be supported by the user is therefore given by (3).

(3) $$\tau_{net}\left(\theta \right)=\tau_{g}\left(\theta \right)-\tau_{p}(\theta )$$

Equation (3) represents the static equilibrium condition at the shoulder joint. The derivation of Equations (1)–(3) is grounded in classical mechanics and biomechanical modeling principles commonly used to analyse passive exoskeleton systems. Equation (1) is derived from Hooke’s law applied to a cable-driven elastic transmission, mapping the spring’s linear deformation to joint rotation via the system geometry. Equation (2) follows from rigid-body dynamics and computes the gravitational torque under the assumption of a lumped mass at the arm segment’s center of mass. Equation (3) represents the static equilibrium condition at the joint, balancing gravitational and assistive torques.

**Figure 4:**
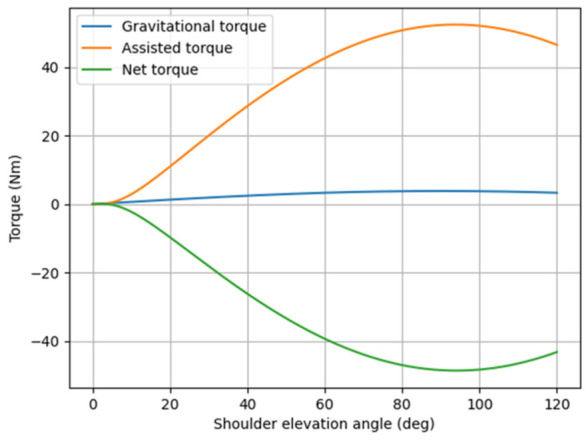
Torque–angle profiles of the shoulder joint.

These equations follow standard biomechanical formulations commonly adopted for passive upper-limb exoskeleton modeling and were adapted to the mechanical configuration of the proposed system. Comparable simplified modeling approaches are widely adopted in computational biomechanics, where reduced-order representations describe complex physical interactions, such as deformation under fluid flow, while maintaining computational tractability [42]. Similar formulations have been widely reported in the literature on passive upper-limb exoskeletons and cable-driven assistive devices, supporting the validity of the adopted modeling approach [43,44]. The comparison between these torque contributions is shown in Figure 4.

The gravitational torque, the passive assistance torque provided by the exoskeleton, and the resulting net torque are plotted as functions of the shoulder elevation angle over the range 0–120°. As shown in Figure 4, the assistance torque increases with shoulder elevation, partially compensating for the arm’s gravitational load. The figure clearly identifies the range of motion in which the exoskeleton is most effective and highlights the residual torque the user must still generate. Boundary conditions and loads are applied to simulate realistic operating conditions. The exoskeleton’s back frame is constrained with a fixed condition to simulate a rigid attachment to the torso, while the shoulder is modelled as a revolute joint aligned with the mediolateral axis. Gravitational acceleration g = 9.81 m/s^2^ is applied to the entire system, and no active torques are applied, ensuring that all joint assistance arises exclusively from the passive elastic elements.

### 2.2. Sensor Instrumentation EEG–EMG Instrumentation and Experimental Protocol

This subsection describes the experimental framework used to acquire multimodal neurophysiological and biomechanical signals during task-oriented motor execution.

A synchronized EEG–EMG recording approach was implemented to capture both central nervous system activity and peripheral muscular responses, enabling a comprehensive characterization of the neuromuscular control underlying human–exoskeleton interaction. The section first describes the sensor instrumentation employed, including the specifications and configuration of the acquisition systems, and then outlines the experimental protocol, encompassing subject recruitment, task design, acquisition parameters, and signal preprocessing procedures. Particular attention is devoted to ensuring temporal synchronization, signal quality, and methodological consistency, which are critical for reliable multimodal data fusion and subsequent analysis.

#### 2.2.1. Sensor Instrumentation

**Figure 5:**
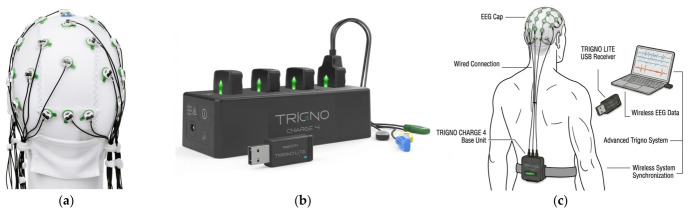
Experimental setup for synchronous EEG–EMG acquisition: (**a**) EEG unit based on the Brain Products LiveAmp^®^ wireless amplifier(Brain Products GmbH, Gilching, Germany) equipped with actiCAP^®^ slim active electrodes(Brain Products GmbH, Gilching, Germany); (**b**) EMG and kinematic acquisition system using the Delsys Trigno Wireless^®^ platform(Delsys Inc., Natick, MA, USA); (**c**) schematic representation of the multimodal EEG–EMG acquisition and processing framework integrated with the passive exoskeleton system.

Synchronous EEG–EMG acquisition was performed during interaction with the passive exoskeleton to capture both central and peripheral components of motor execution (Figure 5). Figure 5 provides a comprehensive overview of the experimental setup, including both the EEG and EMG acquisition systems. Brain activity was monitored using a Brain Products LiveAmp wireless amplifier (24-bit resolution, <130 g), equipped with actiCAP slim active electrodes with high input impedance (>200 MΩ) and low noise (<2 µVpp). The electrodes were integrated into an actiCAP snap cap, providing a compact, stable configuration that minimizes mechanical interference with the exoskeleton. Muscular activity and limb kinematics were acquired using the Delsys Trigno Wireless© system. Trigno Avanti sensors© were employed, providing simultaneous 16-bit surface EMG signals (bandwidth 20–450 Hz) and 9-DOF inertial measurements. The integrated sensor fusion of accelerometer, gyroscope, and magnetometer data enabled real-time estimation of limb orientation using a quaternion representation. Figure 5 shows the visual representation of both acquisition units.

#### 2.2.2. Experimental Protocol

The experimental dataset consisted of 10 healthy adult participants (6 males and 4 females), aged 28 to 46 years (mean ± SD = 36.2 ± 5.4 years). All subjects were right-handed and reported no history of neurological, musculoskeletal, or cognitive disorders. Each subject performed three functional tasks representative of industrial upper-limb activities: Screw, Move Box, and Lift Box, as well as a generic other class that includes transitional or non-task-specific movements. For each task, participants completed three repetitions under five assistive conditions: no exoskeleton, minimum passive assistance, maximum passive assistance, minimum mechanical torque assistance, and maximum mechanical torque assistance, resulting in a total of 45 trials per subject. EEG signals were acquired at an initial sampling rate of 500 Hz and down-sampled to 250 Hz after preprocessing. EMG signals were recorded at 1000 Hz. The sampling rates of the modalities were chosen according to the standard multimodal neurophysiology recording protocols [45]. The EEG was sampled at 500 Hz first, as the raw data needs to be protected from distortions throughout the preprocessing procedure, then down-sampled to 250 Hz for more efficient calculations. The EMG was sampled at 1000 Hz. EEG signals were collected using active wet electrodes, ensuring high signal quality and low impedance. EEG electrodes were placed at F 3, F 4, C 3, and C 4 according to the international 10–20 system, while surface EMG electrodes were positioned bilaterally on the trapezius, middle deltoid, biceps brachii, and triceps brachii muscles following SENIAM guidelines, resulting in a total of eight EMG acquisition channels. All signals were band-pass filtered (EEG: 1–40 Hz; EMG: 20–450 Hz), notch-filtered at 50 Hz to remove power-line interference, and normalized per subject using z-score normalization. Data were segmented into 10 s windows and synchronized with task execution using hardware triggers from the Live Amp system. Although signal analysis was performed using 10 s windows, near-real-time operation was achieved through a partially overlapping sliding-window strategy. This enables continuous updating of predictions at a much higher temporal resolution than the nominal window length. Accordingly, the reported inference latency (<180 ms) refers to the processing time associated with each updated window segment rather than the full acquisition interval. To ensure real-time responsiveness without sacrificing the long-term spectral context required for cognitive load tracking, the data segmentation uses an aggressive sliding-window architecture. Each data matrix contains a full 10 s epoch of historical physiological signals (WL = 10 s), and the window slides forward continuously with a step size (Δ T) of 100 ms (representing a 95% overlap). Consequently, the processing pipeline does not introduce a 10 s operational latency; instead, it ingests new data packets every 100 ms and feeds the updated baseline matrix into the machine learning pipeline. The total computational inference latency of the hybrid CNN–LSTM network, encompassing feature extraction and the forward pass, is strictly bounded below 180 ms, ensuring that the execution loop remains synchronized with the high-frequency demands of dynamic industrial tasks. These windows were used as input samples for the multimodal CNN–LSTM classifier. EEG and EMG signals recorded using the Live Amp system (Brain Products GmbH, Germany) enable precise hardware-level synchronization across multiple biosignal modalities [46]. The selection of a 4-channel configuration (F3, F4, C3, C4) and a 10 s segmentation window was guided by hardware portability and the need for stable spectral estimates of cognitive indices. However, this parameterization introduces specific constraints. Spatially, avoiding central-medial channels (such as Cz) excludes the Supplementary Motor Area (SMA), thereby omitting critical early motor preparation and anticipatory neural potentials. Temporally, while a 10 s epoch ensures stable tracking of slowly varying cognitive fatigue states, it limits real-time responsiveness during highly dynamic, rapid industrial cycles. Consequently, a dual-time-scale approach represents a major structural improvement for the processing pipeline, splitting cognitive monitoring and motor intention classification into distinct, optimized windows. Integrating wearable acquisition platforms with real-time physiological monitoring systems aligns with recent advances in portable biomedical devices for continuous patient monitoring, where multimodal signal acquisition and low-latency processing are essential [47,48]. Accurate hardware-level synchronization and signal integrity are critical for high-speed biomedical acquisition systems and have been extensively addressed in the design of advanced electronic interfaces for low-noise, high-sensitivity signal detection [49]. This configuration, particularly suited for experiments involving naturalistic upper-limb movements, minimizes cable-induced artifacts and constraints on participants’ movements.

**Figure 6:**
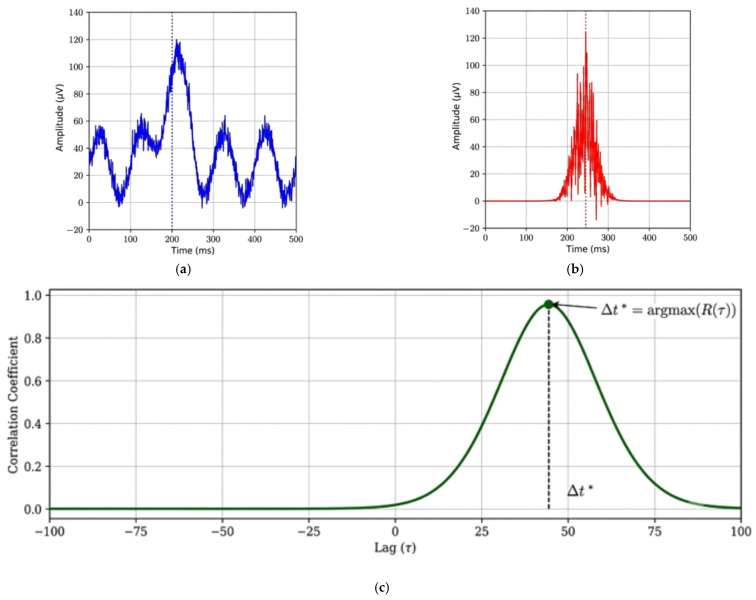
EEG-EMG synchronisation and neuromuscular delay estimation with optimal cortico-muscular latency ∆t*: (**a**) Mean EEG Signal; (**b**) Mean EMG Signal; (**c**) Cross—Correlation Function.

Data segmentation was implemented using a sliding-window approach with partial overlap, enabling near-real-time inference despite the nominal 10 s acquisition window duration [50]. Temporal synchronization between EEG and EMG streams was achieved using hardware triggers and further verified offline through cross-correlation analysis. The optimal delay, denoted ∆t*, is the delay τ that maximizes the cross-correlation between the mean EEG and EMG signals and is illustrated in Figure 6 and given in Equation (4).

Grand average of the cleaned cortical EEG signal (blue) showing initial task-related motor intention peaking at approximately 200 ms, Figure 6a. Grand average of the surface electromyography (sEMG) signal (red) capturing the subsequent peripheral muscle activation burst at approximately 245 ms, Figure 6b. Cross-correlation function R(_T_) (green) computed across a dynamic window, Figure 6c. The absolute maximum peak (green dot) analytically defines the optimal cortico-muscular transmission latency, Equation (4), which is actively utilized by the software-level framework to align multi-sensor data streams and eliminate propagation lag in the real-time classification loop.

(4) $$\Delta t^{*}=\begin{array}{c} argmax\\ \tau \end{array}(xcorr\left(m_{EEG},\;m_{EMG}\right)[\tau ])$$

where $m_{EEG}$ and $m_{EMG}$ denote the mean EEG and EMG signals, respectively. This latency estimate was used to refine multimodal alignment and to inform the interpretation of corticomuscular coupling during task execution. All experimental sessions were conducted using the Ottobock Shoulder X(Ottobock SE & Co. KGaA, Duderstadt, Germany) passive upper-limb exoskeleton.

### 2.3. Multimodal Processing, Adaptive Control, and Performance Evaluation

This subsection outlines multimodal processing for intention recognition from EEG-EMG signals and adaptive assistance for a passive upper-limb exoskeleton.

The approach integrates physiological signal processing, deep learning-based classification, cognitive state assessment, biomechanical assessment, and overall performance assessment within a unified framework. It is important to emphasize that the proposed adaptive framework operates at the interaction and user-state interpretation levels, while the mechanical assistance remains purely passive and unchanged. The control loop formalized in this architecture does not feed back into active physical actuators; rather, it governs a software-level adaptive interpreter. The outputs of the multimodal processing pipeline are used to dynamically update the digital state-space of the human–robot interaction loop.

Accordingly, Section 2.3.1 covers EEG-EMG feature extraction and cognitive indices; Section 2.3.2 presents the CNN-LSTM architecture and multimodal fusion strategies; and Section 2.3.3 addresses performance evaluation, adaptive control logic, and biomechanical evaluation using finite element models.

#### 2.3.1. Feature Extraction

To characterize the user’s cognitive and affective states during task execution, two EEG-derived indices were computed online: the CLI and the FAI. These metrics provide complementary information about mental effort and emotional engagement and are widely used in neuroergonomics and brain–computer interface research [51].

The CLI is defined as the ratio of the spectral power in the theta band to that in the alpha band and reflects the level of mental workload and sustained attention, as shown in Equation (5).

(5) $$CLI=\frac{P_{\theta }}{P_{\alpha }}$$

where *P_θ_* and *P_α_* denote the EEG power in the *θ* (4–7 Hz) and α (8–13 Hz) frequency bands, respectively. Higher *CLI* values indicate greater cognitive demand and prolonged attentional engagement. The **FAI** is used to assess emotional valence and stress-related cortical asymmetries. It is calculated as the logarithmic difference between the alpha-band power recorded at the right (F4) and left (F3) frontal electrodes (Equation (6)).

*FAI* = *log*(*Pα*(*F*4)) − *log*(*Pα*(*F*3)) (6)

Positive *FAI* values are associated with positive emotional engagement and approach-related states, whereas negative values are typically linked to stress, fatigue, or withdrawal-related affect [27]. Both indices are computed in real time and provided to the adaptive assistance module, which modulates the exoskeleton’s support level based on the user’s cognitive and emotional state. The frequency-domain characteristics of the EEG signals were extracted from canonical bands to reflect cognitive engagement, attentional stability, and motor preparation. To complement the previously defined cognitive indices, two additional metrics were introduced at this stage to enhance the adaptive control system’s sensitivity. Neural engagement was quantified using the Neural Engagement Index (*NEI*), defined as the normalized difference between beta-band power during task execution and beta-band power at rest, divided by alpha-band power during rest (7).

(7) $$NEI=\frac{\begin{array}{c} p_{task}\\ \beta \end{array}-\begin{array}{c} p_{rest}\\ \beta \end{array}}{\begin{array}{c} p_{rest}\\ \alpha \end{array}}$$

where $\overset{P_{task}}{\beta }$ denotes the beta-band (13–30 Hz) power measured during task execution, $\overset{P_{rest}}{\beta }$ is the beta-band power during the resting baseline, and $\overset{P_{rest}}{\alpha }$ represents the alpha-band (8–13 Hz) power during the resting baseline. Higher *NEI* values indicate increased cortical activation and task engagement. In addition, a Cognitive–Motor Coupling Index (*CMCI*) was defined to measure the degree of synchronization between neural and muscular activation (8)

(8) $$CMCI=\frac{1}{N}\sum_{k=1}^{N}\left|\rho \left({EEG}_{\beta }^{\left(k\right)},{EMG}_{env}^{\left(k\right)}\right)\right|$$

where $\rho \left({EEG}_{\beta }^{\left(k\right)},{EMG}_{env}^{\left(k\right)}\right)$ is the Pearson correlation coefficient between the beta-band EEG envelope and the EMG envelope over the *k-th* time window, and *N* is the number of windows considered. Larger *CMCI* values indicate stronger coupling between cortical intent and muscular execution, supporting reliable adaptive assistance. These two indices served as complementary high-level descriptors to modulate the assistance profile alongside the previously defined *CLI* and *FAI*, improving robustness in dynamic and cognitively demanding tasks [52]. Time- and frequency-domain EMG features were extracted to characterize muscle activation intensity and fatigue-related spectral variations. The root-mean-square (*RMS*) reported in Equation (9) quantifies the amplitude of muscle activation.

(9) $$RMS=\sqrt{\frac{1}{N}\sum_{n=1}^{N}x{\left(n\right)}^{2}}$$

which provides a reliable estimate of contraction intensity, while signal variability was detected through variance (10)

(10) $$VAR=\frac{1}{N-1}\sum_{n=1}^{N}{\left(x\left(n\right)-\bar{x}\right)}^{2}$$

which reflects fluctuations in muscle recruitment strategies. Fatigue uses the mean frequency (*MNF*), i.e., the spectral centroid of the EMG power distribution, shown in Equation (11).

(11) $$MNF=\frac{\sum_{f}f\;P(f)}{\sum_{f}P(f)}$$

and the median frequency (*MDF*), i.e., the frequency that divides the power spectrum into two regions of equal energy, as shown in Equation (12).

(12) $$\sum_{f\leq f_{MDF}}P\left(f\right)=\frac{1}{2}\sum_{f}P(f)$$

Muscle fatigue during prolonged or repetitive activities is typically associated with a progressive shift in *MNF* and *MDF* toward lower frequencies [53].

To assess the discriminative relevance of features extracted using different multimodal fusion strategies, a nonparametric Friedman test was applied to the classification accuracy results, revealing statistically significant differences among the methods (13).

(13) $$\chi^{2}=37.04\;\;\rho <0.001$$

The Deep Fusion strategy achieved the best overall classification performance. These results confirm that the selected EEG and EMG features provide a robust and statistically validated representation of motor and cognitive states, supporting reliable multimodal gesture recognition and adaptive exoskeleton assistance.

#### 2.3.2. CNN–LSTM Architecture and Multimodal Fusion Strategies

The proposed multimodal classification framework employs a hybrid CNN–LSTM architecture to jointly capture the spatial and temporal characteristics of EEG and EMG signals [54,55]. Separate convolutional branches were adopted for EEG and EMG processing. Each branch consists of two one-dimensional convolutional layers with 32 and 64 filters, a kernel size of 3, and a stride of 1. Each convolutional layer was followed by ReLU activation, batch normalization, and dropout (rate = 0.3) to improve generalization and reduce overfitting. The extracted feature maps were subsequently provided to an LSTM layer with 64 hidden units to model temporal dependencies across consecutive signal windows. The final hidden state of the LSTM is then fed into a fully connected layer with SoftMax activation to produce class probabilities over the four gesture classes.

**Figure 7:**
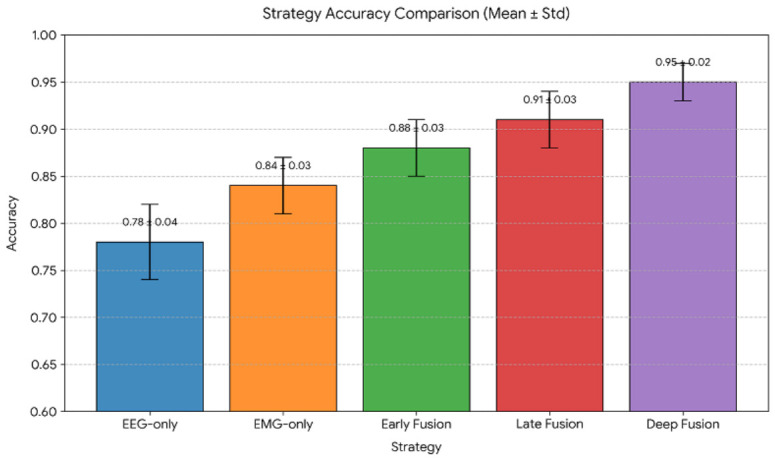
Ablation Study—Classification Accuracy.

The network is trained using the Adam optimizer with a learning rate of 10^−3^ and categorical cross-entropy as the loss function. Training is performed for 100 epochs with a batch size of 32. A stratified subject-independent 5-fold cross-validation strategy was adopted to improve robustness and reduce the risk of subject-specific overfitting. To rigorously assess the generalization capabilities of the hybrid CNN–LSTM architecture on unseen operators, a Leave-One-Subject-Out (LOSO) cross-validation framework was implemented. Given the user cohort of N = 10, the model underwent 10 independent training and evaluation cycles. In each fold, data from N-1 subjects constituted the training set, while the remaining subject was entirely withheld to serve as the independent testing baseline. Crucially, because the feature extraction pipeline uses overlapping sliding windows, special countermeasures were implemented to eliminate the risk of temporal data leakage. Data splitting was performed strictly at the subject and experimental-trial levels prior to window segmentation and data augmentation. Consequently, adjacent or overlapping temporal frames were confined within their respective subject boundaries. Furthermore, z-score normalization parameters (mean and variance) were computed exclusively on the training subset and subsequently applied to the test subset, ensuring that no statistical descriptors of the test subject’s neurophysiological profile were introduced during the model’s training phase. Model selection was based on the average F1-score across validation folds. To investigate the contribution of multimodal integration, an ablation study was conducted by comparing three fusion strategies, namely Early Fusion, Late Fusion, and Deep Fusion, in addition to unimodal EEG-only and EMG-only baselines. The results of the ablation study are summarized in Table 2 and presented in Figure 7. In the early fusion strategy, feature embeddings extracted from EEG (he) and EMG (hm) signals are concatenated before temporal modeling, forming a joint multimodal sequence (14).

(14) $$u=[h_{e}|\left|h_{m}\right]\;,\;\;\;\;\;\;\;\;g=LSTM(u_{1:T})$$

This approach captures cross-modal relationships early but lacks adaptive weighting mechanisms. Late Fusion operates at the decision level by combining the outputs of unimodal classifiers via a weighted sum (15).

(15) $$\hat{y}=\alpha \hat{y_{e}}+(1-\alpha )\hat{y_{m}}$$

where $\hat{y_{e}\;}and\;\hat{y_{m}}$ denote the EEG- and EMG-based predictions, respectively, and α ∈ [0, 1] controls their relative contribution. The most advanced strategy, Deep Fusion, introduces an adaptive gating mechanism within the network. After temporal modeling, the EEG and EMG latent representations, *g_e_* and *g_m_*, are concatenated and processed by trainable fusion layers (16) and (17).

(16) $$a=\sigma (W_{a}\left[h_{e}|\left|h_{m}\right]+b_{a}\right),$$

(17) $$g=a⊙g_{e}+\left(1-a\right)⊙g_{m}$$

where σ(·) denotes the sigmoid activation function and ⊙ denotes the Hadamard product. This formulation enables adaptive context-dependent weighting of each modality, improving robustness under noisy or dynamically varying conditions.

Detailed performance metrics are summarized in Table 2, while Figure 7 provides a visual comparison of ranking accuracy across strategies.

The Deep Fusion strategy achieved an accuracy improvement of approximately 11–17% compared with unimodal approaches. Hyperparameter optimization was performed through randomized search combined with stratified 5-fold cross-validation.

**Figure 8:**
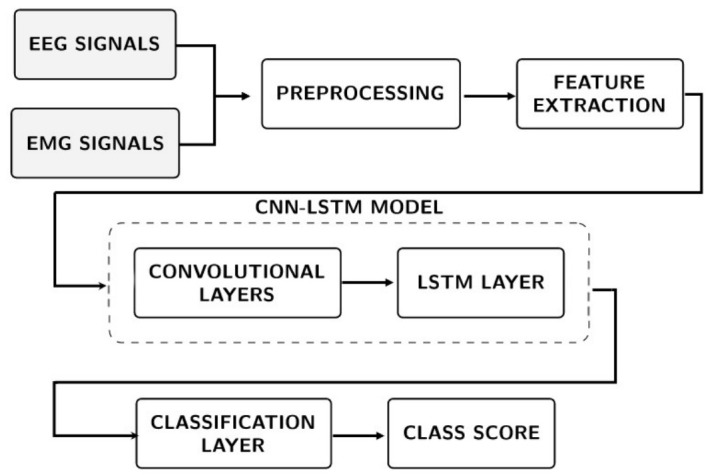
CNN–LSTM Model Pipeline.

Figure 8 illustrates the overall processing pipeline of the proposed hybrid CNN–LSTM framework.

The diagram summarizes the transformation of EEG–EMG signals into multimodal feature representations, fusion layers, and adaptive classification outputs for exoskeleton interaction analysis.

The processing pipeline also highlights the sequential integration of convolutional feature extraction, temporal dependency modeling, and adaptive multimodal fusion, enabling robust intention recognition and cognitively informed interaction with the passive exoskeleton system.

#### 2.3.3. Performance Metrics, Adaptive Control, and Biomechanical Interpretation

Model performance uses standard classification metrics, including accuracy, defined in Equation (18), precision, recall and F1 score.

(18) $$Accuracy=\frac{TP+TN}{TP+TN+FP\prime +FN^{\prime }}$$

while precision and recall are given by (19)

(19) $$Precision=\frac{TP}{TP+FP\prime }\;\;\;\;\;\;Recall=\frac{TP}{TP+FN\prime }$$

The F1 score, defined in Equation (20), is the harmonic mean of precision and recall.

(20) $$F1=2\cdot \frac{Precision\cdot Recall}{Precision+Recall}$$

Inference latency (21) represents a critical performance indicator for near-real-time wearable applications. It is quantified as the median processing time required by the model to generate a prediction for each incoming signal window, with variability reported using the interquartile range (IQR).

(21) $$L_{inf}=median\;(t^{predicion}-t^{input})$$

To compare the fusion strategies statistically, a non-parametric Friedman test was applied. The test revealed statistically significant differences among the evaluated methods.

(22) $$\chi^{2}=24.36\;\;\;\;\;\rho <0.001$$

The statistical analysis (22) confirmed significant performance differences among the evaluated fusion strategies, with Deep Fusion achieving the best overall results.

Post hoc pairwise comparisons using the Nemenyi test were additionally performed to identify statistically significant differences among fusion strategies. Beyond gesture classification, cognitive-state estimation was integrated into the adaptive assistance interpretation framework.

The assistance provided by the exoskeleton is modelled as expressed in Equation (23).

(23) $$u\left(t\right)=u_{0}\left(\hat{y}\right)\cdot g(CLI,FAI)$$

where $u_{0}\left(\hat{y}\right)$ represents the reference torque associated with the recognised gesture $\hat{y}$. The assistance term in Equation (23) does not represent externally actuated torque generation. Instead, it describes the equivalent biomechanical contribution associated with the passive elastic assistance provided by the exoskeleton, whose interaction effects are interpreted through the proposed adaptive physiological framework without introducing external energy into the system.

The cognitive gain g(·) is indicated in Equation (24)

(24) $$g=1+k_{1}{\left[CLI-\tau_{CLI}\right]}^{+}-k_{2}{\left[\tau_{FAI}-FAI\right]}^{+}$$

Equation (24) increases assistance under high cognitive load and reduces it when stress or fatigue is present. The proposed adaptive framework was developed according to safety-oriented design principles consistent with ISO 13482 recommendations for personal care robots. Conservative limits, smoothing filters, and fallback strategies ensure stable operation under abnormal physiological or mechanical conditions.

Biomechanical interpretation, supported by finite element modeling, approximates the assistance torque generated by the elastic elements in passive exoskeletons using Equation (25).

(25) $$\tau_{p}\approx \;k_{s}\Delta l\left(\theta \right)r(\theta )$$

where *k_s_* is the elastic constant, Δ*l(θ)* is the cable elongation, and *r(θ)* is the effective lever arm. By contrast, active exoskeletons generate torque according to Equation (26).

(26) $$\tau_{a}\left(\theta \right)=K(\theta ,\dot{\theta },\;\ddot{\theta })$$

thereby enabling continuous torque modulation across the joint range of motion.

To maximize operational safety and mitigate the effect of classification uncertainties during transitional phases, a Reject-Option (or Null-Class) mechanism is embedded into the decision layer of the hybrid CNN–LSTM framework. Let x represent the multimodal window input, and let $\hat{P}(y=c|x)$ denote the posterior probability distribution output by the final Softmax layer across all defined task classes $c\in \{C_{1},C_{2},\ldots ,\prime \mathrm{Other}\prime \}$. A confidence threshold Γ∈[0,1] is formally established. The final control command Y^*^ is governed by the following conditional mapping:

(27) $$Y^{*}=\left\{\begin{array}{c} \arg{max}_{c}\hat{P}(y=c|x),\;\;\;\;\;\;\;\;\;\;if\;{\;max}_{c}\hat{P}(y=c|x)\geq \Gamma \;\\ NullState,\;\;\;\;\;\;\;\;\;\;\;\;\;\;\;\;\;\;\;\;\;\;\;\;\;\;\;if{\;\;max}_{c}\hat{P}(y=c|x)<\Gamma \end{array}\right.$$

When the maximum posterior probability falls below Γ, the intent is deemed ambiguous, triggering a ‘Null State’. In this state, the software-level adaptation suspends any active tracking parameters and maintains baseline passive ergonomics, ensuring that low-confidence transitional movements do not trigger false-positive assistance profiles.

FEM-based analysis provides quantitative information on load redistribution and ergonomic benefits, complementing physiological and control-oriented assessments.

Overall, the proposed framework integrates multimodal EEG–EMG acquisition, deep learning-based fusion strategies, cognitive-state estimation, and FEM-assisted biomechanical interpretation within a unified passive exoskeleton architecture. The methodology was designed to support near-real-time operation, physiological interpretability, and biomechanical consistency while preserving the intrinsic safety and mechanical simplicity of passive assistive systems.

#### 2.3.4. Conceptual Architecture for Real-Time FEM-Informed Control

To extend the utility of the biomechanical model beyond offline validation, a real-time FEM-informed control logic is conceptually introduced. Because full-scale non-linear FE simulations are computationally prohibitive for online applications, the offline parametric FEM dataset is utilized to train a Reduced-Order Surrogate Model (ROM).

Operating within the real-time execution loop, this surrogate model continuously ingests the classified task context from the CNN–LSTM pipeline and the current joint kinematics. It instantaneously outputs the structural stress distribution and the theoretical muscle relief torque. In active or semi-active variants of this framework, this real-time FEM output serves as a feedforward control signal to dynamically adjust actuator gains or physical spring-tension weights, aligning the robotic assistance with physics-verified ergonomic targets.

#### 2.3.5. EMG-Driven Software-Based Auto-Calibration Routine

To mitigate the long-term effects of mechanical spring degradation without requiring external sensor load cells, a software-based auto-calibration routine is introduced. Let $EMG_{RMS}^{c}(t)$ be the moving average of the root-mean-square EMG envelope for a specific classified task *c* at shift *t*, and let $EMG_{RMS}^{c}(0)$ represent the nominal baseline recorded during initial system commissioning. A structural degradation index $\alpha_{\deg}$ is continuously updated via a long-term smoothing filter:

(28) $$\alpha_{deg}=\frac{1}{K}\sum_{k=1}^{K}\frac{EMG_{RMS}^{c}(t-k)}{EMG_{RMS}^{c}(0)}$$

where K represents the integration window spanning multiple work cycles. A persistent increase in *α_deg_* > 1.0 under identical kinematic trajectories (extracted from joint angles) indicates that the physical spring stiffness has degraded, forcing the user to recruit additional muscle fibers to compensate for the loss of passive torque. The software architecture utilizes this index to adaptively rescale its internal ergonomic thresholds, adjusting the user’s fatigue assessment profiles and triggering automated digital maintenance logs when *α_deg_* surpasses a critical safety threshold. The experimental results presented in the following section evaluate classification performance, multimodal fusion effectiveness, cognitive adaptation mechanisms, and biomechanical assistance characteristics across different task conditions.

## 3. Results

The multimodal model presented is characterised by a comprehensive combination of numerical simulations, experimental data, and prototype-based validation. The objective is to evaluate classification performance, cognitive adaptability, biomechanical consistency, and real-time feasibility under realistic operating conditions. The simulation results confirmed the stability of the CNN-LSTM architecture with deep EEG-EMG fusion under controlled perturbations, including additive noise, channel imbalance, and partial signal degradation. To address the sensitivity of FEM results to boundary conditions, experimental repeatability was assessed across multiple trials and participants. The consistency of the observed torque–angle trends across different assistive conditions indicates that the model-predicted mechanical behavior is reproducible and robust. Although the FEM model is not intended for precise quantitative prediction, the agreement between simulated trends and experimental observations supports its validity as a qualitative biomechanical reference. The system maintained robust classification performance while keeping inference latency below the real-time threshold required for wearable robotic applications across all tested scenarios. Compared with unimodal strategies and non-adaptive fusion approaches, Deep Fusion consistently provided superior performance, owing to its ability to dynamically weight neural and muscular information in the presence of disturbances and task variability. The experimental protocol consisted of ten healthy adult subjects (six males and four females). The subjects were recruited according to stringent inclusion criteria to exclude any neurological, muscular, or cognitive disorders. The participants, all right-handed, performed functional tasks with their upper limbs without limitations. Each subject completed forty-five experimental trials, comprising three functional tasks (screwing, moving a box, and lifting a box), five assistance conditions (no exoskeleton, minimum and maximum passive assistance, minimum and maximum semi-active assistance), and three repetitions for each task. Rest intervals were introduced to minimise fatigue-related effects and preserve signal quality. The EEG and EMG signals, acquired synchronously during the performance of the activity, were segmented into ten-second windows aligned with the motor activity using synchronised markers. The adequacy of the sample size, verified by power analysis (α = 0.05, power = 0.8, Cohen’s d ≈ 0.5), ensures sufficient statistical power. The CNN-LSTM model has shown high, consistent classification performance across all gesture classes. As shown in Table 3, the best accuracy was obtained for the screwing activity (0.92). Moreover, good classification accuracy was also obtained for box moving and lifting. For the diverse “Other” class, accuracy was slightly compromised but remained within acceptable limits. The mean macro accuracy and F1-score are also high at 0.88 and 0.82, respectively.

**Figure 9:**
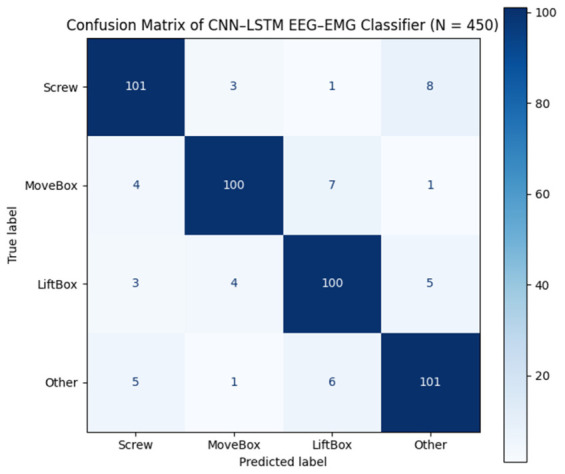
Confusion matrix of the CNN–LSTM classifier showing gesture classification performance across all experimental tasks.

The lower classification accuracy observed for the ‘Other’ class reflects the inherent kinematic and physiological variability of untargeted transitional movements. However, the introduction of the conditional Reject-Option threshold Γ strategically isolates the human–robot interaction loop from these classification errors. By funnelling all predictions that yield high entropy or low softmax margins into a safe ‘Null State’, the system effectively trades off a negligible fraction of operational throughput for a substantial increase in system deterministic safety. This prevents the exoskeleton software from erroneously locking into a task-specific assistance mode when the user is simply performing unstructured intermediate manoeuvres. The confusion matrix in Figure 9 further confirms the model’s generalization ability across tasks, with classification errors occurring mainly between dynamically similar movements.

The “Other” class achieved an F1-score of 0.82. To mitigate false-positive activations during transitional movements, a confidence threshold and temporal smoothing strategy were applied.

**Figure 10:**
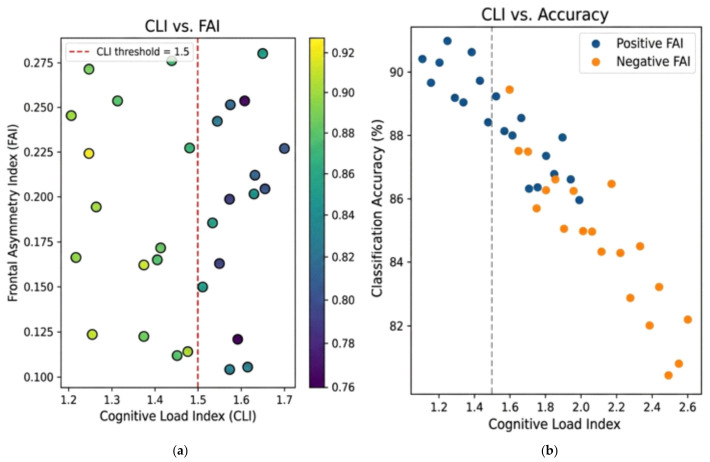
Correlation between cognitive indices and classification accuracy: (**a**) Cognitive Load Index (CLI) versus accuracy; (**b**) Frontal Asymmetry Index (FAI) versus accuracy.

The relationship between neurophysiological indices and classification performance is shown in Figure 10. The system maintains approximately 90% accuracy when the CLI remains below the 1.5 threshold. The value obtained is largely independent of the FAI. Conversely, high CLI values, which are associated with a progressive reduction in accuracy, indicate stress or fatigue, particularly when FAI values are negative.

A positive FAI can partially counteract the effect of increased cognitive load and help maintain classification reliability. This demonstrates that multimodal EEG-EMG fusion not only improves the reliability of motor recognition but also enables the incorporation of cognitive and affective information for adaptive assistance.

EEG–EMG recordings were made simultaneously during task execution, with time annotations for data segmentation. Each trial was recorded in 10 s intervals, and start and end markers were synchronized with motor activity.

**Figure 11:**
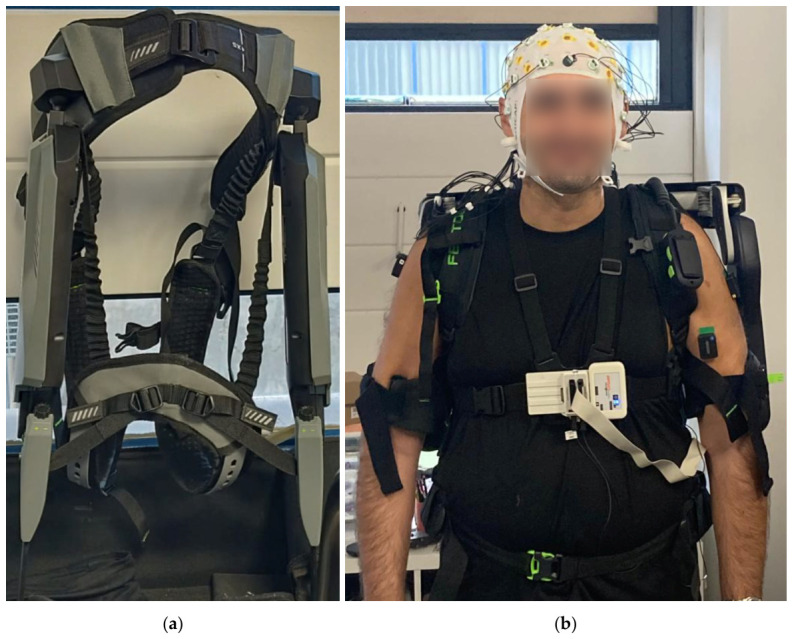
Passive exoskeleton prototype: (**a**) Structure; (**b**) Upper limb prototype worn by a healthy subject during experimental validation.

Figure 11 highlights the mechanical structure, shoulder interface, elastic assistance mechanism, and attachment points used to transfer biomechanical loads during functional activities. Beyond algorithmic performance, biomechanical validation was conducted by comparing finite element method (FEM) simulations with experimental measurements from the passive exoskeleton prototype worn by a healthy subject.

**Figure 12:**
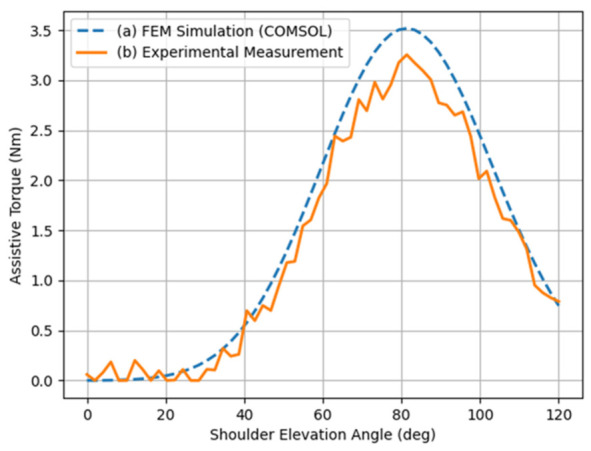
Comparison between assistive torque profiles obtained from FEM-based COMSOL Multiphysics 5.2© simulations (a) and experimental measurements on the passive exoskeleton prototype (b) as a function of shoulder elevation angle.

Figure 12 reports the assistive torque profiles as a function of shoulder elevation angle. The FEM-based COMSOL Multiphysics 5.2© simulation shows an ideal spring-like characteristic, with the highest level of assistance in the mid-range of motion (60–90°). The experimentally determined torque profile closely matches the simulated trend, with a slightly reduced amplitude due to mechanical compliance, transmission losses, and measurement uncertainties. The high degree of correspondence between the experimentally determined torque curve and the FEM-based simulation result substantiates the validity of the FEM model and confirms the prototype’s conformity with the expected biomechanical principles.

The experimentally measured torque, shown in Figure 12, follows the same trend as the simulated torque, though it peaks lower due to mechanical compliance, transmission losses, and measurement uncertainty. This agreement confirms the validity of the FEM model and shows that the prototype behaves as expected in line with biomechanical principles. When comparing passive and active exoskeletons, a clear trade-off between simplicity and flexibility emerges. Passive exoskeletons provide maximum assistance in mid-range postures, are suitable for prolonged or constant activities, require no external power supply, and offer high energy efficiency. In contrast, active exoskeletons provide continuous, programmable torque across the entire range of motion, making them better suited to dynamic tasks and high-load conditions.

**Figure 13:**
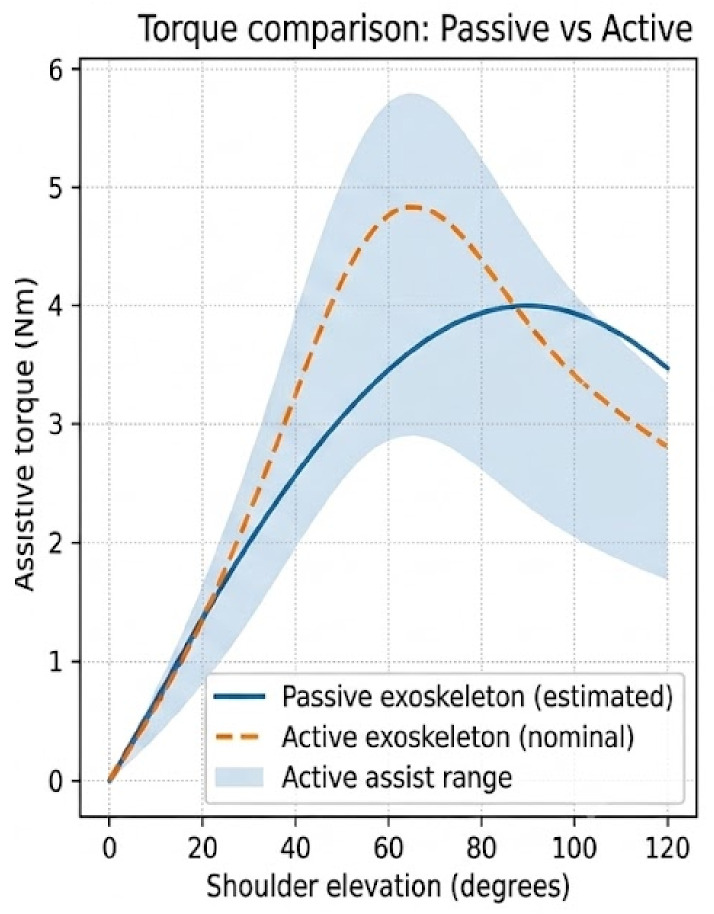
Comparison of FEM-simulated and experimentally measured assistance torque as a function of shoulder elevation angle. The FEM model predicts a spring-like behavior with peak assistance in the mid-range (60–90°), which closely matches the prototype measurements.

Figure 13 clearly illustrates the fundamental differences between passive and active assistance strategies. The passive exoskeleton exhibits spring-like behavior, with torque concentrated in the mid-range of motion, which is optimal for quasi-static or repetitive tasks requiring energy-efficient support. In contrast, the active system provides programmable, continuous torque across the full range of motion, enabling higher peak values and dynamic modulation to match task demands and user intent. This comparison confirms the complementary nature of the two approaches and supports the use of cognitively adaptive control to select and tune the appropriate assistance profile based on the operational context. Overall, the results demonstrate that the proposed EEG–EMG-based multimodal framework achieves reliable real-time classification, meaningful cognitive adaptation, and biomechanical consistency, as validated by FEM simulations and prototype measurements. These findings establish a solid experimental foundation for intelligent, safe, and interpretable neural interfaces for wearable robotics and naturally motivate the broader considerations and future perspectives discussed in the following section.

## 4. Discussion

From algorithmic, cognitive, and biomechanical perspectives, the data obtained in the above section indicates the success of the introduced framework. This section presents the analysis of the major findings, their practical application prospects for passive exoskeleton devices, as well as existing limitations and research directions in this field.

### 4.1. Multimodal Classification Performance

This paper presented an integrated multimodal framework for adaptive assistance in passive upper-limb exoskeletons, combining synchronous EEG–EMG acquisition, deep learning-based fusion, cognitive state estimation, and finite element method (FEM)-assisted biomechanical modeling within a single experimental and computational architecture. The proposed method is designed with a focus on applicability, real-time capability, and interpretability, which are primary requirements in assistive technology used in ergonomic and industrial settings. From a signal processing and machine learning perspective, the experimental results demonstrate that the proposed CNN–LSTM architecture with Deep Fusion achieves reliable gesture classification under realistic operating conditions, reaching a macro-average accuracy of 0.90 and a macro-average F1-score of 0.85, with an inference latency below 180 ms, thus fully satisfying real-time application requirements. Ablation studies demonstrate the superiority of the proposed method, showing a significant performance gap between unimodal and multimodal approaches and yielding a 15-percentage-point reduction in error. This performance gain highlights the complementary nature of EEG and EMG signals, in which neural information enhances anticipatory intent detection, while muscular activity provides robustness against motion artifacts and inter-subject variability. An additional contribution of this study is the integration of neurophysiological indices related to cognitive state, namely the CLI and the FAI. The experimental evidence indicates that these indices are meaningfully correlated with classification performance and can be used to support adaptive modulation of assistance.

In particular, the system maintains stable performance under moderate cognitive load, while excessive mental effort and unfavorable affective states lead to a gradual.

Degradation in accuracy. From an application standpoint, this behavior enables the assistive system to adapt its support strategy to the user’s neurophysiological state, mitigating excessive cognitive or emotional stress without compromising task execution.

The practical benefit of this software-level framework is best illustrated in a dynamic factory use case where an operator alternates between overhead screwing and tool-retrieval tasks. While standard passive devices penalize the user during downward movements by requiring antagonistic muscle activation to overcome the springs, the proposed multimodal architecture flags these phases using the ‘Other’ task class and cognitive strain metrics. This state awareness provides a measurable ergonomic benefit, ensuring that integrating passive mechanics does not come at the cost of secondary physical resistance or an elevated mental workload. In addition, the observed link between the Frontal Asymmetry Index (FAI) and classification accuracy suggests the possibility of developing user-specific cognitive training methods that can be implemented in a neurocognitive system through neurofeedback, stress management techniques, and cognitive resilience training programs, among other options. By helping users maintain positive frontal asymmetry during exoskeleton operation, the described cognitive training methods will increase the likelihood of maintaining favorable human cognitive states. Although such cognitive training methods have not been evaluated in the current study, they warrant investigation in future research.

### 4.2. FEM-Based Biomechanical Validation

In addition to algorithmic validation, the study includes a biomechanical validation stage comparing FEM simulation results with empirical measurements from a passive exoskeleton prototype worn by a healthy subject. The FEM simulations predicted a spring-like assistive torque profile, peaking in the mid-range of shoulder elevation (60–90°). The experimental measurements show a similar trend, although with a reduced amplitude attributable to mechanical compliance and transmission losses. The close correspondence between numerical predictions and experimental measurements strongly validates the FEM-based biomechanical model and confirms the suitability of the proposed prototype as a reliable design and assessment tool for passive assistive devices.

### 4.3. Implications for Passive Exoskeleton Systems

A comparative evaluation of passive and active exoskeleton approaches offers additional insight into the advantages and limitations of the proposed concept relative to specific task requirements and target populations, including industrial workers and patients undergoing rehabilitation. Introducing additional IoT-based devices would enable predictive assessment of long-term safety and reliability. In this context, the proposed cognitively adaptive framework enhances the functional effectiveness of passive exoskeletons without altering their fundamental mechanical nature, representing a pragmatic and scalable solution for a wide range of applications. It should be emphasized that the proposed adaptive assistance does not increase the exoskeleton’s mechanical torque. The system remains entirely passive, and adaptation is limited to software-level interpretation of physiological and cognitive information. Consequently, the primary advantage over conventional passive devices lies in enhanced context awareness, fatigue monitoring, task interpretation, and support optimization rather than in active biomechanical torque generation.

### 4.4. Actionable Neurofeedback Training (NFT) for Operator Resilience

To translate the protective effects of a positive FAI state into industrial practice, a structured Neurofeedback Training (NFT) protocol is recommended for onboarding operators. Because frontal alpha asymmetry reflects an individual’s approach-related motivation and capacity for emotional regulation, users can be trained to actively maintain a positive FAI profile. Before factory-floor deployment, operators undergo brief, closed-loop biofeedback sessions in which the real-time FAI stream, computed directly from the F3 and F4 channels, is mapped to an intuitive graphical user interface (GUI) or to an auditory modulation tone delivered via wearable industrial headsets. Operators are instructed to apply cognitive approach strategies, such as target-oriented mindfulness and focus-rejuvenation techniques. When an operator achieves a positive FAI state, the system provides positive sensory reinforcement. Integrating this physics and neuro-informed training within corporate ergonomic workflows provides an actionable pathway to cultivate cognitive resilience, ensuring that operators can naturally buffer against mental workload spikes associated with complex, high-velocity human–robot collaboration tasks.

### 4.5. Mechanism of the Auto-Calibration Strategy

While mechanical wear and spring relaxation remain physical realities for passive systems, the proposed EMG-driven auto-calibration routine transforms this hardware limitation into a software-monitored variable. By tracking cumulative muscle payload trends over extended operational periods, the digital control layer can pinpoint the onset of mechanical degradation. This physics- and muscle-informed tracking mechanism significantly extends the system’s operational longevity, transforming a standard, non-communicating passive device into an intelligent, self-diagnosing wearable asset capable of predicting its own maintenance needs.

### 4.6. Limitations

While the proposed multimodal framework demonstrates high classification accuracy and stable cognitive tracking, certain limitations must be acknowledged. First, the experimental validation was conducted on a restricted cohort of ten healthy subjects (N = 10). While this sample size is mathematically and statistically sufficient for pilot methodological validation, it does not fully capture the physiological and kinematic diversity of the primary target end-users, such as older adults or professional industrial workers. Aging and chronic occupational physical stress significantly alter muscle firing rates, co-contraction dynamics, and EEG microstates. Furthermore, the ‘Other’ task class, representing transitional and non-assisted movements, was evaluated under semi-structured laboratory conditions. In a live factory setting, this class is highly variable, encompassing unstructured activities such as interacting with control panels, walking on uneven surfaces, or resting. Future field studies will focus on deploying this architecture in active manufacturing environments, expanding the cohort to include industrial operators across varying age groups, and enriching the deep learning dataset with authentic, unstructured workplace movements to ensure long-term algorithmic robustness and generalization.

Additionally, the reduced number of EEG channels represents a trade-off between practical usability and spatial resolution, potentially limiting the ability to discriminate among highly complex tasks. While this simplified montage improves wearability and deployment feasibility, it does not directly capture activity in the supplementary motor area (SMA), which contributes to motor planning and movement preparation. Future studies may investigate expanded electrode configurations to assess potential improvements in detecting anticipatory intention. Furthermore, the algorithmic framework exhibits an intrinsic trade-off between system responsiveness and classification confidence. Shifting from the current 10 s epoch to shorter windows of 2–5 s is essential for adapting the system to high-speed industrial environments. Shortening the window inherently reduces the number of data points per sample, which can hinder the convergence of deep learning architectures, such as hybrid CNN–LSTM networks. To resolve this trade-off without sacrificing ergonomic efficacy, future architectures will decouple the multimodal pipeline: a fast, responsive branch operating on a 2 s sliding window will handle kinematic and muscular movement intentions, while a parallel, slower-integrating branch will continuously compute cognitive workloads. This optimization, combined with the inclusion of an additional EEG node over the SMA, will unlock highly responsive, anticipatory robotic control while preserving robust neurophysiological metrics. Similar trade-offs between spatial resolution and practical usability have been reported in wearable EEG systems designed for real-world applications, where minimal electrode configurations are preferred to improve user comfort and deployment feasibility [56,57]. Moreover, the cognitive indices derived from EEG features were defined empirically and require further clinical validation. From a deployment perspective, replacing lab-grade wet electrodes with industrially viable alternatives is imperative. Although this study validated the adaptive control logic with a stable electrode setup, scaling this solution to real-world manufacturing floors requires transitioning to dry-contact sensors or in-ear EEG nodes. Dry electrodes mitigate the physical degradation of the conductive medium during extended hours of physical labor. In-ear EEG devices offer a minimally invasive, highly ergonomic alternative that integrates naturally with the auditory protection devices already mandatory in noisy industrial environments, effectively addressing both signal reliability and operator compliance over long-term deployments [58,59]. From a hardware perspective, the current prototype lacks integrated sensors for long-term mechanical diagnostics, such as stress, temperature, or interface pressure monitoring.

Another fundamental challenge for long-term industrial translation is the temporal degradation of classifier performance caused by cumulative user fatigue. Over an 8 h work shift, progressive physical exhaustion slows muscle fiber conduction velocity (typically reflected in a downward shift in the EMG power spectrum’s median frequency), while mental fatigue triggers persistent increases in alpha-band power and theta-band variations in the EEG. Because static deep learning architectures, such as the proposed hybrid CNN–LSTM model, are trained on non-fatigued baseline data, these physiological non-stationarities constitute a covariate shift that can severely degrade task classification accuracy over time. To insulate the human–robot loop from this degradation, the framework’s concurrent monitoring of the Cognitive Load Index (CLI) and Fatigue Assessment Index (FAI) serves as a critical supervisory layer. When the FAI or CLI crosses pre-established individual fatigue thresholds, the software-level architecture can switch from static inference mode to a fatigue-compensated state. Future iterations of this work will explore online adaptive mechanisms, such as continual learning loops and real-time domain adaptation algorithms, allowing the CNN–LSTM network to dynamically update its internal feature weights throughout the shift without requiring disruptive recalibration sessions. Furthermore, long-term degradation of elastic components, such as springs, may alter the torque–angle relationship, reducing assistance accuracy and necessitating periodic recalibration.

## 5. Conclusions

This study presented an integrated multimodal framework for adaptive assistance in passive upper-limb exoskeletons, combining synchronous EEG–EMG acquisition, deep learning-based fusion, cognitive-state estimation, and finite element method (FEM)-assisted biomechanical modeling within a unified experimental and computational architecture. The proposed approach was designed to improve the functionality of passive assistive systems while preserving their intrinsic safety, simplicity, and energy efficiency.

The experimental results demonstrated that multimodal EEG–EMG fusion, implemented through the proposed CNN–LSTM architecture with Deep Fusion, enables reliable gesture classification under realistic operating conditions, achieving a macro-average accuracy of 0.90 and a macro-average F1-score of 0.85 with an inference latency below 180 ms. Furthermore, the integration of cognitive-state indicators provides additional physiological information that can support cognitively informed adaptation strategies. Notably, the proposed adaptive assistance does not involve active torque generation; rather, adaptation is achieved through software-level interpretation of neurophysiological and biomechanical information while maintaining the exoskeleton’s fully passive nature.

The agreement between numerical simulations and prototype measurements confirms the validity of the FEM-based biomechanical model and supports its use as a practical design and assessment tool for passive assistive devices. Overall, this study demonstrates that integrating multimodal EEG–EMG fusion, deep learning, cognitive state estimation, and FEM-based biomechanical modeling is an effective approach to improving the functionality of passive upper-limb exoskeletons. The consistency between numerical simulations and prototype measurements, together with robust real-time classification and cognitively informed adaptation, provides a solid applied foundation for developing intelligent, safe, and ergonomically optimized assistive systems.

Future work will extend experimental validation to larger, more heterogeneous populations, including industrial operators and rehabilitation patients. Integrating additional IoT-based sensors will support predictive monitoring and long-term safety assessment. Although the adopted 10 s windows provided robust classification performance, future investigations will systematically evaluate shorter window durations (2–5 s) to quantify the trade-off between classification accuracy and responsiveness in high-speed industrial scenarios. A major avenue for future research is transitioning the FEM component from an interpretive offline diagnostic tool to an online control primitive. By embedding the proposed FEM-trained surrogate model in the hardware control layer, the system can bypass the limitations of purely passive mechanics. When integrated with active or semi-active actuators, this real-time FEM-informed logic will enable adaptive physical weight adjustments, dynamically scaling the assistance profile based on instantaneous biomechanical stress predictions rather than static lookup tables, thereby maximizing ergonomic offloading across highly volatile industrial workflows. Furthermore, the proposed multimodal fusion strategy may be extended to semi-active or active exoskeletons, enabling a unified control framework across actuation paradigms while complying with relevant safety standards, such as ISO 13482 [60]. Overall, this study demonstrates that integrating multimodal EEG–EMG fusion, deep learning, cognitive state estimation, and FEM-based biomechanical modeling is an effective approach for improving the functionality of passive upper-limb exoskeletons. The consistency between numerical simulations and prototype measurements, together with robust real-time classification and cognitively informed adaptation, provides a solid applied foundation for developing intelligent, safe, and ergonomically optimized assistive systems.

## Figures and Tables

**Table 1:** Mechanical properties assigned to exoskeleton components in the FEM model.

Component	Material Type	Elastic Modulus	Source
Back frame (a)	Aluminium alloy	~70 GPa	ASM Handbook
Main side arm (c)	Aluminium alloy	~70 GPa	Engineering standards
Connection interface (b)	Fiber-reinforced polymer	12 GPa	Composite literature
Spring housing (d)	Fiber-reinforced polymer	10–15 GPa	Manufacturer data
Arm interface (e)	Fiber-reinforced polymer	10–15 GPa	Manufacturer data

**Table 2:** Results of the ablation study comparing unimodal and multimodal fusion strategies.

Strategy	Accuracy (Mean ± SD)	Precision (Mean ± SD)	Recall (Mean ± SD)	F1-Score (Mean ± SD)
EEG-only	0.78 ± 0.04	0.76 ± 0.05	0.79 ± 0.05	0.77 ± 0.04
EMG-only	0.84 ± 0.03	0.83 ± 0.04	0.85 ± 0.03	0.84 ± 0.03
Early Fusion	0.88 ± 0.03	0.87 ± 0.03	0.89 ± 0.03	0.88 ± 0.03
Late Fusion	0.91 ± 0.03	0.90 ± 0.03	0.91 ± 0.02	0.90 ± 0.02
Deep Fusion	0.95 ± 0.02	0.94 ± 0.02	0.96 ± 0.02	0.95 ± 0.01

**Table 3:** Performance metrics by class for the CNN–LSTM classifier.

Class	Accuracy	Precision	Recall	F1-Score
Screw	0.92	0.91	0.88	0.89
Movement box	0.91	0.84	0.89	0.86
Lift box	0.89	0.86	0.82	0.84
Other	0.88	0.80	0.85	0.82
Macro-average	0.90	0.85	0.86	0.85

## Data Availability

The original contributions presented in this study are included in the article. Further inquiries can be directed to the corresponding author.
